# A Deep Learning Model for Accurate Maize Disease Detection Based on State-Space Attention and Feature Fusion

**DOI:** 10.3390/plants13223151

**Published:** 2024-11-09

**Authors:** Tong Zhu, Fengyi Yan, Xinyang Lv, Hanyi Zhao, Zihang Wang, Keqin Dong, Zhengjie Fu, Ruihao Jia, Chunli Lv

**Affiliations:** China Agricultural University, Beijing 100083, China; zhutong22@cau.edu.cn (T.Z.); yanfengyi@cau.edu.cn (F.Y.); lvxinyang@cau.edu.cn (X.L.); zhaoyihan2023@cau.edu.cn (H.Z.); wangzihang23@cau.edu.cn (Z.W.); keqin23@cau.edu.cn (K.D.); zhengjiefu24@cau.edu.cn (Z.F.); jrh24@cau.edu.cn (R.J.)

**Keywords:** maize leaf disease detection, state-space attention mechanism, deep learning in agriculture, image-based disease identification, computer vision for plant health

## Abstract

In improving agricultural yields and ensuring food security, precise detection of maize leaf diseases is of great importance. Traditional disease detection methods show limited performance in complex environments, making it challenging to meet the demands for precise detection in modern agriculture. This paper proposes a maize leaf disease detection model based on a state-space attention mechanism, aiming to effectively utilize the spatiotemporal characteristics of maize leaf diseases to achieve efficient and accurate detection. The model introduces a state-space attention mechanism combined with a multi-scale feature fusion module to capture the spatial distribution and dynamic development of maize diseases. In experimental comparisons, the proposed model demonstrates superior performance in the task of maize disease detection, achieving a precision, recall, accuracy, and F1 score of 0.94. Compared with baseline models such as AlexNet, GoogLeNet, ResNet, EfficientNet, and ViT, the proposed method achieves a precision of 0.95, with the other metrics also reaching 0.94, showing significant improvement. Additionally, ablation experiments verify the impact of different attention mechanisms and loss functions on model performance. The standard self-attention model achieved a precision, recall, accuracy, and F1 score of 0.74, 0.70, 0.72, and 0.72, respectively. The Convolutional Block Attention Module (CBAM) showed a precision of 0.87, recall of 0.83, accuracy of 0.85, and F1 score of 0.85, while the state-space attention module achieved a precision of 0.95, with the other metrics also at 0.94. In terms of loss functions, cross-entropy loss showed a precision, recall, accuracy, and F1 score of 0.69, 0.65, 0.67, and 0.67, respectively. Focal loss showed a precision of 0.83, recall of 0.80, accuracy of 0.81, and F1 score of 0.81. State-space loss demonstrated the best performance in these experiments, achieving a precision of 0.95, with recall, accuracy, and F1 score all at 0.94. These results indicate that the model based on the state-space attention mechanism achieves higher detection accuracy and better generalization ability in the task of maize leaf disease detection, effectively improving the accuracy and efficiency of disease recognition and providing strong technical support for the early diagnosis and management of maize diseases. Future work will focus on further optimizing the model’s spatiotemporal feature modeling capabilities and exploring multi-modal data fusion to enhance the model’s application in real agricultural scenarios.

## 1. Introduction

In modern agricultural production, precise disease detection and management are key factors in ensuring healthy crop growth and increasing yield [1,2,3]. Particularly for maize, a widely cultivated staple crop, timely and effective disease detection is crucial not only for the efficiency and economic benefits of agricultural production but also because it directly impacts global food security [4,5,6]. Thus, developing an efficient maize leaf disease detection technology has significant practical application value.

Traditional disease detection methods primarily rely on manual visual identification, which is not only inefficient but also subject to the influence of the inspectors’ experience and subjective judgment, leading to inaccuracies in disease diagnosis [7,8]. Jamjoom et al. [9] found that manually detecting and treating plant diseases is highly challenging. They used an image processing system based on SVM to detect diseases affecting plants capable of identifying and classifying four different forms of plant diseases, including Phytophthora, Fusarium graminearum, Puccinia graminis, and tomato yellow leaf curl virus. According to experimental data, this technique can correctly detect and diagnose plant diseases with an accuracy rate of 0.972. However, these algorithms mostly require manual feature extraction, limiting the model’s generality and scalability.

With the rapid development of information technology, computer vision has been introduced into the field of disease detection, automating disease recognition through the processing and analysis of digital images [10,11,12]. However, early computer vision systems often relied on simple image processing techniques, such as color segmentation and morphological operations, which have poor robustness in complex environments and struggle to cope with the diversity of natural lighting and backgrounds [13]. In recent years, deep learning technology has made significant progress in image recognition; in particular, convolutional neural networks (CNNs) have become a powerful tool for processing visual tasks [14]. Pramudhita et al. [15] proposed a system capable of using CNNs based on leaf images for high-accuracy disease classification to detect diseases in strawberry plants. The proposed system is a CNN algorithm using MobileNetV3-Large and EfficientNet-B0 models to train a preprocessed four-class dataset. Using these architectures helps to keep the number of parameters and model size minimal. The study’s results showed that the MobileNetV3-Large model performed better than EfficientNet-B0. The best accuracy, which was achieved using the MobileNetV3-Large architecture and RMSProp hyperparameter optimizer with 70 epochs and a learning rate 0.0001, reached 0.9214. The evaluation model using MobileNetV3-Large achieved a precision, recall, and F1 score values of 0.9281, 0.9214, and 0.9225, respectively. However, the model’s dataset is imbalanced, and the CNN’s local feature extraction often falls short in complex scenarios, necessitating a shift to models capable of global contextual understanding. By refining and adjusting the ViT (Vision Transformer) model, Rachman et al. [16] revealed its exceptional ability, surpassing that of traditional CNN models (including VGG, MobileNet, and EfficientNet). The proposed ViT model achieved an accuracy of 0.98 on the dataset, demonstrating superior performance and stability across different tasks and datasets.

Although deep learning models exhibit superior performance in disease detection, these models often require extensive labeled data and have limitations in processing time-series data and spatial transformations. In response, Chen et al. [17] proposed a transformer model based on cycle-consistent generative adversarial networks for generating diseased tomato leaf images for data augmentation. Additionally, they used the transformer model and densely connected CNN architecture to extract multi-level local features. The experiments showed that the proposed model achieved an accuracy of 0.9945 on the PlantVillage dataset. However, the model’s robustness still needs further verification to adapt to complex and variable field environments. Brown et al. [18] found that traditional detection methods have inherent drawbacks, including time-consuming processes, labor-intensive work, and the need for expert knowledge. They improved agricultural practices by using cutting-edge classification algorithms, combining CNNs and ViT to enhance accuracy and efficiency. Experimental results confirmed the superior performance of the advanced hybrid ViT model, with an accuracy of 0.8886. Zeng et al. [19] proposed a two-stage model called DIC-Transformer, which contains three tasks (detection, interpretation, and classification). In the first stage, using a Swin transformer as the backbone, Faster R-CNN detects disease areas and generates feature vectors of diseased images. In the second stage, the model uses the transformer to generate image captions. Then, an image feature vector weighted by text features is generated to enhance the performance of image classification in the subsequent classification decoder. Results show that DIC-Transformer outperforms other comparative models in classification and caption generation. However, existing deep learning methods often overlook the contextual relationships and long-distance dependencies in disease detection, limiting their application effects in complex scenarios.

These studies demonstrate the potential and challenges of deep learning in complex agricultural scenarios. In particular, the disease issues of maize, as one of the world’s key staple crops, are notably severe [20]. Common diseases such as northern corn leaf blight and southern rust not only impact the efficiency of photosynthesis in maize leaves but can also lead to significant yield reductions. The frequency and scale of maize diseases necessitate more precise monitoring and management to avert potential food crises [21,22]. Symptoms typically include spots, yellowing, and deformities on the leaves. Zhang et al. [3,12] proposed a CNN based on a multi-pathway activation function module and the TinySegformer model for high-precision detection of corn leaf diseases and agricultural pests, respectively. In facing these challenges, the application of computer vision and deep learning technologies for early detection and classification of maize diseases can significantly enhance accuracy and efficiency, which are crucial for global food production.

In light of this, this article proposes a maize leaf disease detection model based on a state-space attention mechanism, aiming to overcome the limitations of traditional methods and improve detection accuracy and efficiency. The main innovations of this article include the following:

- State-space attention mechanism: In traditional deep learning models, although CNNs can effectively recognize image features, they often overlook the temporal information and spatial relationships in images, especially in dynamic changes during disease evolution. For this reason, we designed an innovative state-space attention mechanism. This mechanism not only focuses on the local features of maize leaves but also captures the temporal evolution and spatial distribution of leaf diseases through the state-space model. Through this method, the model can finely perceive the subtle changes from the initial to development stage of the disease, significantly improving the recognition ability in early stages of the disease, which is crucial for early intervention and management of the disease.
- State-space function: The state-space function is introduced to more efficiently adjust and optimize the weight distribution in the attention mechanism. Traditional attention mechanisms are often fixed or change little, making it difficult to adapt to complex and variable practical application scenarios. Our state-space function dynamically adjusts weights according to the real-time health condition of the maize leaves, allowing the model to adaptively adjust its focus according to the specific development stage and type of disease. This adaptive adjustment can greatly enhance the model’s accuracy in recognizing different disease states in practical applications, thereby more precisely determining the type and development trend of the disease.
- State loss function: To optimize the performance of the state-space attention mechanism, we developed a new loss-function–state-loss function. This loss function is specifically designed for the characteristics of disease detection and can effectively reduce the occurrences of misdiagnosis and missed diagnosis during the disease detection process. By weighting the prediction errors in different states, this loss function can more accurately guide model learning, optimizing the model’s generalization ability and robustness in complex agricultural environments, thereby improving the overall detection accuracy.

Through these innovative technologies and methods, this study not only enhances the technological level of maize leaf disease detection but also provides a reliable technological path and practical application cases for the realization of precision agriculture and intelligent agriculture, showcasing the broad application prospects of deep learning technology in modern agriculture.

## 2. Related Work

### 2.1. Transformer

Since its introduction in 2017 [23], the transformer model has rapidly achieved remarkable success in various fields, such as natural language processing and computer vision [24,25,26,27]. Its core lies in the self-attention mechanism and multi-head attention mechanism, which enable the transformer to efficiently capture long-range dependencies in sequence data.

The basic principle of the self-attention mechanism is to dynamically adjust the importance of elements within the input sequence by calculating relationships between them [28,29]. In this mechanism, for a given input ($x_{i}$), attention scores are computed using three representations: Query, Key, and Value. First, the input is mapped into Query, Key, and Value through linear transformations:

(1) $$Q=W_{q}\cdot X,\;K=W_{k}\cdot X,\;V=W_{v}\cdot X$$

where $W_{q}$, $W_{k}$, and $W_{v}$ are learnable weight matrices, and *X* is the input sequence. Next, the attention scores are obtained by calculating the similarity between the Query and all Keys, usually through a dot product operation:

(2) $$\mathrm{Attention}\left(Q,K,V\right)=\mathrm{softmax}\left(\frac{QK^{T}}{\sqrt{d_{k}}}\right)V$$

where $d_{k}$ is the dimensionality of the Key vector used to scale the dot-product values and avoid overly large values that could lead to gradient vanishing or explosion. By applying the softmax function, normalized attention weights are obtained, which are then used to compute a weighted sum of the values, resulting in the final output. This mechanism allows the model to focus on the important parts of the input and, in image processing, to efficiently extract features. In the field of computer vision, ViT divides an image into fixed-size patches and treats these patches as sequence data for processing [30]. ViT captures the relationships between patches through the self-attention mechanism, enhancing its ability to model global contextual information. Compared to traditional convolutional neural networks, ViT can handle inputs of any size and, due to its parallel computing capabilities, significantly speeds up training.

In disease detection tasks, the advantages of the transformer are particularly prominent. By introducing a transformer, we can better handle complex structures and features in images, especially in natural environments where disease manifestations are often diverse and difficult to predict. The transformer, through its powerful feature extraction capabilities, dynamically adjusts its attention to different regions of the image, allowing for more effective identification of key information such as lesions and discoloration [31,32]. For example, when small lesions appear on maize leaves, traditional methods may struggle to detect them effectively, but with the transformer, the model can focus on areas around the lesion through the self-attention mechanism, improving detection accuracy. Additionally, the transformer’s advantage in handling sequential data allows it to track the progression of diseases and detect changes and spread in a timely manner.

### 2.2. MAMBA

MAMBA (Multi-Modal Attention-Based Analysis) is a multi-modal attention analysis framework developed in recent years, designed to enhance feature learning and task performance by fusing data from different modalities (e.g., images, text, and sensor data) [33]. This framework has shown its powerful advantages in the field of agriculture, particularly in crop health monitoring and disease detection tasks [34,35]. By combining spectral data, image data, and meteorological information, MAMBA improves the model’s adaptability to environmental changes, thereby enhancing the accuracy and robustness of disease detection.

The core idea of MAMBA is to use the attention mechanism to dynamically weight data from different modalities [36,37]. Its basic principle is to process each modality’s data through the self-attention mechanism, calculating its importance within the overall information. For the input data from multiple modalities ($x_{i}$), MAMBA also uses the Query, Key, and Value representations, computing attention weights using these representations.

Next, MAMBA calculates the attention scores between different modalities to dynamically adjust the weighting of the information. The calculation of attention scores is similar to traditional self-attention mechanisms. MAMBA then performs a weighted summation of the attention scores across multiple modalities to obtain a fused representation. This mechanism allows the model to automatically adjust the focus between different input modalities, extracting more representative features.

In agricultural disease detection, MAMBA’s advantages are mainly reflected in the following aspects. First, agricultural data are typically diverse and complex, and a single modality of data may not sufficiently express the crop’s health status [38]. By fusing image data, sensor data, and meteorological information, MAMBA can comprehensively consider various influencing factors, providing a more complete analysis. Secondly, MAMBA’s dynamic weighting feature enables the model to adjust its focus when facing complex backgrounds and environmental changes. For example, when monitoring a specific disease, the model can automatically enhance its attention to modalities related to the disease and reduce interference from irrelevant information [39,40].

### 2.3. Attention Mechanism

The attention mechanism is a key technique in deep learning, designed to dynamically adjust the weights of input features, allowing the model to focus more on critical parts when processing information. This mechanism was initially widely applied in natural language processing, but with the development of the technology, its applications have expanded to computer vision, speech recognition, and other fields [41,42,43]. In image recognition tasks, the attention mechanism can help models effectively focus on important regions in the image, significantly improving classification and detection performance [44,45].

The basic principle of the attention mechanism is to dynamically adjust the weights of input features according to their importance. When processing input data, the model first calculates the relevance of each feature to determine its importance. Specifically, for a given input feature vector ($x_{i}$), the attention mechanism generates attention weights by calculating Query, Key, and Value. Query represents the feature that needs attention, Key is the feature used for matching with others, and Value corresponds to the actual information associated with the Key.

In image processing tasks, the attention mechanism can be seen as a feature selection process. When the model receives an image input, the attention mechanism helps to identify the most relevant regions for the target task. For example, in disease detection, the model can prioritize regions with lesions or discoloration, ignoring irrelevant background information. This characteristic makes the attention mechanism particularly effective in complex visual tasks [46,47].

In our research, the maize leaf disease detection model based on the state-space attention mechanism leverages the advantages of attention mechanisms to enhance detection accuracy and efficiency. By incorporating the state-space model, the proposed method can dynamically capture changes in features during disease progression, improving the model’s sensitivity to diseases.

## 3. Materials and Method

### 3.1. Materials

#### 3.1.1. Dataset Collection

In this study, our goal is to develop a maize leaf disease detection model based on a state-space attention mechanism. To achieve this goal, constructing the dataset is a crucial step. This section provides a detailed description of the data collection process, including the locations, equipment, methods, and disease characteristics, ensuring the scientific rigor and effectiveness of the study. First, the data collection was divided into three parts: field collection, online image acquisition, and data preprocessing. We selected two main collection sites: the Science Park on the West Campus of China Agricultural University in Haidian District, Beijing, and Fuqiang Village in Linhe District, Bayannur City, Inner Mongolia. These locations are representative and can reflect the characteristics of maize diseases in different environments. Maize plants in the Science Park grow in a controlled environment, making them suitable for experimental observation. Meanwhile, Fuqiang Village in Bayannur City is a typical maize-growing region, allowing us to collect real samples from natural conditions. In this study, we utilized a Canon EOS 90D camera for photography, primarily equipped with a standard lens. The reason for choosing a standard lens lies in its exceptional versatility, enabling it to adeptly cater to the requirements of most shooting scenarios. This camera was equipped with a high-performance lens that can capture clear images under varying lighting conditions. Additionally, we used a tripod to stabilize the camera during shooting, avoiding any image blurring caused by shaking. During the image capture process, special attention was paid to changes in lighting, and all photos were taken on sunny days to minimize the impact of shadows on image quality. When selecting maize plants for image collection, we targeted those at suitable growth stages. In the Science Park, we marked plants at different growth stages, such as the seedling stage, vegetative growth stage, and maturity stage. Each growth stage included representative disease samples to ensure the diversity and comprehensiveness of the data. In Bayannur City, we chose healthy maize fields to ensure the quality of the collected images. Furthermore, during field observations, we also recorded environmental factors such as temperature, humidity, and wind speed, as these factors could influence disease presentation. For the collection of maize leaf disease images, we focused on six major diseases: maize northern leaf blight, maize southern leaf blight, maize smut, maize head smut, maize round spot, and maize brown spot. The number of images for each disease ranged from 800 to 1700, ensuring sufficient data for subsequent training and testing, as shown in Table 1.

**Figure 1:**
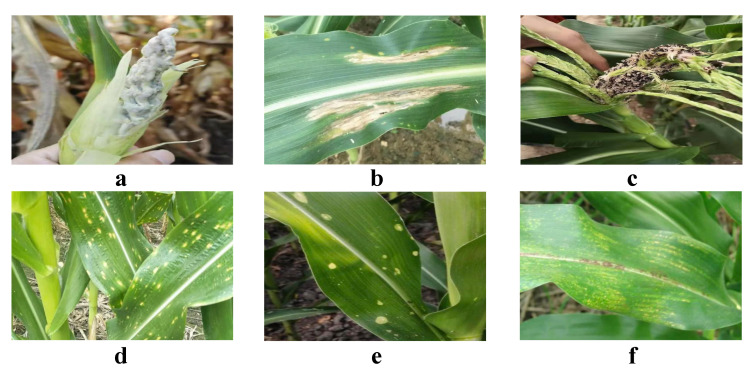
Dataset samples. (**a**) Maize head smut; (**b**) maize northern leaf blight; (**c**) maize smut; (**d**) maize southern leaf blight; (**e**) maize round spot; (**f**) maize brown spot.

As illustrated in Figure 1, the characteristic symptoms of maize northern leaf blight include large, brown lesions with blurred edges on the leaf surface, often accompanied by leaf shrinkage and yellowing. When collecting images of this disease, we focused on capturing these typical symptoms and recorded the size, shape, and distribution of the lesions. We ensured that each photo clearly showed the disease characteristics for subsequent analysis. Maize southern leaf blight is characterized by smaller brown spots on the leaves, often with distinct yellow halos around the edges. To ensure data diversity, we took images of this disease at various growth stages and under different environmental conditions. During the collection process, we also paid particular attention to distinguishing between northern and southern leaf blight so that the model could accurately differentiate these two diseases in subsequent analyses. Maize smut and maize head smut show significant differences in appearance. Maize smut primarily presents as black lesions on the ears, while head smut forms distinctive tumor-like structures on the plant. When collecting images of these diseases, we recorded detailed observations to ensure that all disease details were captured. Moreover, the characteristics of maize round spot and maize brown spot are relatively complex. Maize round spot manifests as circular lesions, often with clear edge changes, while brown spot appears as irregular brown patches. When capturing images of these diseases, we paid special attention to photographing the details of different lesion shapes for thorough analysis. After field collection, we also utilized online resources to obtain additional images of maize diseases to enrich the diversity of the dataset.

#### 3.1.2. Data Augmentation

**Figure 2:**
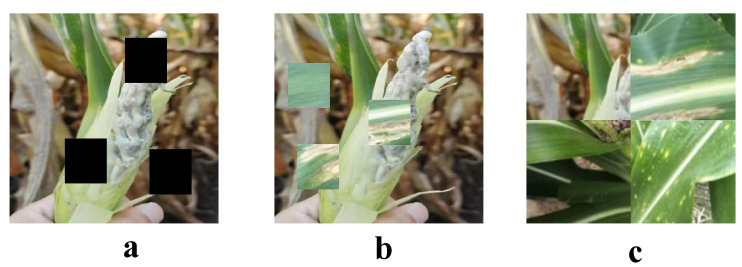
Dataset augmentation. (**a**) CutOut; (**b**) CutMix; (**c**) Mosaic.

To further improve the model’s generalization ability, we applied several data augmentation techniques during dataset construction. These techniques not only effectively prevent model overfitting but also enhance the model’s adaptability to environmental changes, thereby improving the accuracy of disease detection. In practice, we used some augmentation methods, such as CutOut, CutMix, and Mosaic, as shown in Figure 2.

CutOut is a technique that enhances model robustness by obscuring part of the image. Specifically, we randomly selected a rectangular area in the image and set it to a background value (usually 0 or the mean value). The formula is expressed as

(3) $$I^{\prime }=I\cdot \left(1-M\right)+B\cdot M,$$

where *M* is a binary mask of the same size as the image, with a value of 1 indicating the obscured area, and *B* is the background value. The CutOut technique effectively prevents the model from over-relying on specific features, thereby improving the model’s ability to handle unknown samples. CutMix is an augmentation technique that creates new samples by blending two images ($I_{1}$ and $I_{2}$). This blending is not solely based on a linear combination but involves a mask (*M*) that defines how pixels from $I_{1}$ and $I_{2}$ are combined. Specifically, a binary mask (*M*) is created, where some regions are set to 1 and others are set to 0. The new image is then generated using the following formula:

(4) $$I^{\prime }=M⊙I_{1}+\left(1-M\right)⊙I_{2}$$

where ⊙ denotes element-wise multiplication. Correspondingly, the label for the new sample is adjusted based on the area proportion of *M*, denoted as λ, which is calculated as the sum of all ones in *M* divided by the total number of pixels in *M*. The new label is calculated as follows:

(5) $$y^{\prime }=\lambda y_{1}+\left(1-\lambda \right)y_{2}$$

This approach allows the model to learn from a richer set of features by experiencing a diverse combination of characteristics from both images, enhancing the model’s detection capabilities and generalization potential. The use of a mask (*M*) allows for more dynamic and spatially varying patterns of feature integration compared to simple linear blending, providing robust training enhancements. Mosaic is a technique that stitches multiple images together to generate new ones. In this way, we can generate new samples while preserving features from each image and combining their labels for enhanced data diversity. The basic idea of the Mosaic operation is to stitch four different images together into one large image. The expression is

(6) $$I^{\prime }=\left[\begin{array}{cc} I_{1} & I_{2}\\ I_{3} & I_{4} \end{array}\right]$$

The Mosaic technique can significantly increase the diversity of the dataset and enhance the model’s comprehensive understanding of different features. By applying a combination of the above data augmentation techniques, we can effectively expand the size of the dataset and improve the model’s adaptability to environmental changes.

#### 3.1.3. Dataset Construction

During dataset construction, we paid particular attention to the balanced distribution of samples for each disease type to avoid the impact of class imbalance. Class imbalance often leads to model overfitting on certain categories while lacking recognition ability for others. Therefore, we ensured that the number of samples for each disease type was relatively balanced, providing adequate representation for each class. Additionally, we conducted cross-validation during the dataset construction process. Specifically, we divided the entire dataset into training, validation, and test sets, with the training set accounting for about 0.7, the validation set for 0.2, and the test set for 0.1. In summary, we successfully constructed a comprehensive dataset that includes various types of disease images.

### 3.2. Proposed Method

#### 3.2.1. Overall

**Figure 3:**
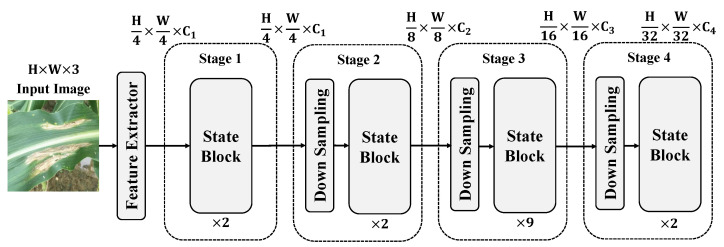
The overall workflow diagram of the proposed corn leaf disease detection model based on a state-space attention mechanism. Here, *H* and W represent the height and width of the input image, respectively, while $C_{i}$ represents the number of channels in the output feature maps at different stages of the model. $C_{1}$, $C_{2}$, $C_{3}$, and $C_{4}$ correspond to the channel dimensions at stages 1, 2, 3, and 4, respectively. Each ’state block’ processes the feature maps, and ’downsampling’ reduces dimensions and increases the channel depth at subsequent stages.

In this paper, we construct a multi-stage feature extraction and fusion model based on a state-space attention mechanism for precise detection of maize leaf diseases. The model begins with the processed input images, which are first passed through a feature extraction module to extract initial features. These features are then further explored and fused through multiple stages of state-space modules, as shown in Figure 3.

The processed images are initially fed into the feature extraction module. The main function of this module is to extract low-level features from the original input image, performing preliminary convolution operations and activations to obtain feature maps. These feature maps contain basic information such as edges, textures, and colors of the image, providing a foundation for subsequent processing by the state-space modules.

The extracted feature maps are gradually sent to the multi-stage state-space modules, where each stage consists of several state blocks. These state blocks are responsible for modeling the spatiotemporal relationships in the features to capture the spatial distribution and temporal dynamics of diseases. Overall, the model consists of four stages: In stage 1, the input feature maps are passed through two consecutive state blocks, which perform local feature capture and state updates on the input features. At this stage, the size of the features remains $\frac{H}{4}\times \frac{W}{4}\times C_{1}$. The main objective is to extract local features from the image and perform preliminary state modeling to capture spatial variations. After processing through stage 1, the feature maps move to stage 2, which also includes two state blocks. The feature size remains the same as in stage 1, that is, $\frac{H}{4}\times \frac{W}{4}\times C_{1}$. This stage further delves into the local features of the image, enabling the model to capture more detailed textures and edge information, thereby enriching the feature representation. Before entering stage 3, the feature maps undergo a downsampling operation to reduce the spatial dimensions of the feature maps and enhance the abstraction capability of the features. The downsampling reduces the feature map size from $\frac{H}{4}\times \frac{W}{4}$ to $\frac{H}{8}\times \frac{W}{8}$ while increasing the number of channels to $C_{2}$, allowing the features to better capture global information during subsequent state-space modeling. In stage 3, the feature maps are processed through nine consecutive state blocks, where each state block performs deeper spatiotemporal relationship modeling on the features. Due to the downsampling operation, the feature dimensions are further reduced to $\frac{H}{8}\times \frac{W}{8}\times C_{2}$. The primary purpose of this stage is to extract and abstract higher-level features from the image, providing a larger receptive field that enables the model to better understand complex disease patterns globally. After stage 3, the feature maps undergo another downsampling operation, reducing the dimensions to $\frac{H}{16}\times \frac{W}{16}\times C_{3}$, then proceed to stage 4. Stage 4 contains two state blocks aimed at capturing higher-level semantic information and further integrating global features. Finally, after the last downsampling operation, the feature dimensions are reduced to $\frac{H}{32}\times \frac{W}{32}\times C_{4}$. Within each state block, the state-space attention mechanism plays a crucial role. Each state block uses state-space functions to model spatial and state information in the feature maps, combining features from different locations with temporal dynamics. The state blocks consider both the current features and the preceding states to generate new feature representations, which are then used for subsequent state updates.

Through the processing of these stages, the model can fully extract features from diseased regions in the input image, model spatial relationships, and capture temporal dynamics. This provides a rich feature basis for subsequent detection and classification tasks. In summary, the proposed model achieves multi-layered, multi-scale deep learning from input images to disease features through staged state-space modules and multi-scale feature extraction strategies, offering an efficient feature representation and analysis framework for maize leaf disease detection.

#### 3.2.2. State-Space Attention Mechanism

**Figure 4:**
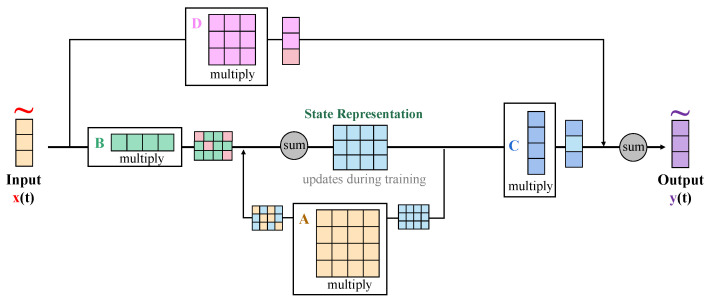
Illustration of the state-space attention mechanism.

The state-space attention mechanism proposed in this paper is an improvement on the traditional transformer self-attention mechanism, designed to better handle the spatiotemporal features in the task of maize leaf disease detection, as shown in Figure 4.

Unlike the original transformer self-attention mechanism, the state-space attention mechanism not only focuses on the global relationships among features but also models the temporal dynamics of these features to capture the changes and connections between different states in the development of diseases. The state-space attention mechanism introduces the concept of state space, where the features are updated at each time step (*t*), and the state representation is used to dynamically model the features. Compared to the self-attention mechanism in transformers, state-space attention not only considers spatial information but also incorporates temporal information during state updates, enabling the model to handle multi-stage changes in the development of diseases more flexibly. The design of the state-space attention mechanism includes three main parts: feature input, state update, and output generation. Each state block includes the following parameters and operations:

1. Input features (x(t)): The input is the feature representation at each time step (*t*), with dimensions of H×W×C, where *H* and *W* represent the height and width of the feature map and *C* is the number of channels.
2. State representation ($\tilde{B}$): The state-space model introduces the state representation ($\tilde{B}$) based on the input features to capture spatiotemporal information. The state representation is updated at each time step according to the input features.
3. State update: The state update process involves a series of matrix operations, including multiplication and summation. The specific update process is as follows: First, the input features (x(t)) are transformed by the state matrix ($\tilde{B}$) to generate state information. The state matrix ($\tilde{B}\left(t\right)$) is defined as $\tilde{B}\left(t\right)=F\left(S\left(t-1\right),x\left(t-1\right)\right)$, where *F* is a function that updates the state matrix based on the previous state (S(t−1)) and the input at the previous time step (x(t−1)). This function facilitates learning of the dynamics between the evolving state and the new input features. Then, the state information is multiplied by the weight matrix (*A*) and combined with the previous state information through a weighted sum to obtain the new state representation. The mathematical expression is as follows:
  (7) $$\tilde{S}\left(t\right)=A\cdot x\left(t\right)+\tilde{B}\left(t-1\right)$$
  where $\tilde{S}\left(t\right)$ is the state representation at time *t*, *A* is the state-space transformation matrix, and $\tilde{B}\left(t-1\right)$ is the state information from time t−1. Through this update mechanism, the model can effectively capture changes along the temporal dimension, accounting for both the influence of prior states and transitions influenced by previous inputs.
4. Output generation: After the state update is completed, the model generates the output (y(t)) and further processes the state information. The calculation of the output is as follows:
  (8) $$y\left(t\right)=C\cdot \tilde{S}\left(t\right)+D\cdot x\left(t\right)$$
  where *C* and *D* are learnable weight matrices. By performing a weighted summation of the state information ($\tilde{S}\left(t\right)$) and the input features (x(t)), the final output features (y(t)) are generated.

This multi-stage structure design allows the model to deeply extract features and perform state modeling at each stage, enhancing its ability to capture features at different scales and temporal variations. The introduction of the state-space attention mechanism improves the model’s ability to capture spatiotemporal information because it closely combines input features with state information, forming dynamically updated state representations. The proof is as follows:

1. Modeling capability in the temporal dimension: During the state update process, the model uses the multiplication and summation operations of matrices *A* and $\tilde{B}$ to model the associations between the input features at time *t* and the states from previous time steps. Thus, this mechanism can capture changes in the input features over time.
2. Feature fusion and context awareness: By performing weighted summation of the state representation and input features through matrices *C* and *D*, the state-space attention mechanism achieves a fusion of local features and global context, further enhancing the model’s representation capability.

In summary, the state-space attention mechanism effectively improves the model’s understanding and representation of spatiotemporal relationships without significantly increasing computational complexity. Therefore, by applying the state-space attention mechanism to maize leaf disease detection, the model demonstrates excellent detection performance in experiments, accurately identifying diseases and capturing their development dynamics.

#### 3.2.3. State-Space Function

First, the input feature (x(t)) is transformed by the state matrix ($\tilde{B}$), which is a learnable weight matrix updated during model training to capture the nonlinear relationship between the input features and the state. Then, the generated state information is fused with the state information from the previous time step (t−1, $\tilde{B}\left(t-1\right)$).

Through the effect of these weights, the model effectively combines feature information and state information to generate a more representative output feature (y(t)). The design of the state-space function takes into account the correlation between features and states in the temporal dimension. Through the mathematical structure mentioned above, it achieves a dynamic fusion of input features and state information. First, the introduction of the state matrix ($\tilde{B}$) enables the model to remember and capture the state characteristics from time step t−1, providing continuity and dynamic perception capability in the temporal dimension. Secondly, through the weighted processing by matrices *A* and *C*, the model can adaptively adjust the strategy for state updates based on the current input feature (x(t)). This design allows the state-space function to dynamically adjust the feature weights during different stages of disease progression, making the model more sensitive to key states and effectively improving the accuracy of disease detection. Furthermore, in the task of maize leaf disease detection, the development of the disease is a dynamic process that can be influenced by various factors, such as light conditions, lesion spread speed, and leaf structure characteristics. Traditional attention mechanisms often struggle to sufficiently model these spatiotemporal relationships. In contrast, the state-space function comprehensively processes both features and states, enabling the model to capture long-range dependencies and temporal variations in disease development. This design not only achieves unified modeling of features and states mathematically but also enhances the model’s robustness and generalization ability for disease detection.

#### 3.2.4. Multi-Scale Fusion Module

**Figure 5:**
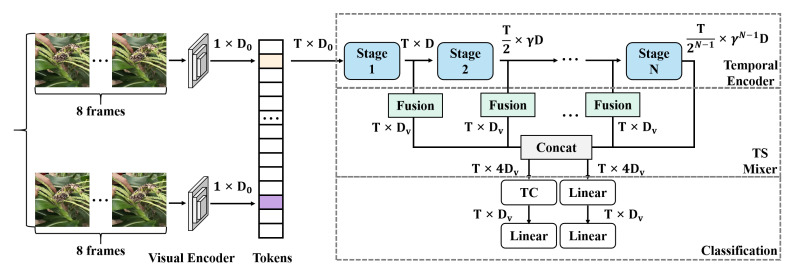
Illustration of the multi-scale fusion module. The module employs “Temporal Convolution” (TC) to process time-series data merged through multiple stages, extracting features along the temporal dimension. The final classification step includes two distinct processes: “TC —> Linear” where features processed by temporal convolution undergo a linear transformation, and “Linear —> Linear” which represents a sequential linear transformation to enhance feature integration before classification. Further details on TC and the rationale behind the dual linear transformations are provided in the figure annotations.

The primary role of the multi-scale fusion module is to extract and fuse features at different scales to capture the spatial and scale diversity of disease features, as shown in Figure 5.

The purpose of this module is to fully utilize information from different spatial scales of the input image while ensuring the model’s precision and robustness. Below is the detailed design of the multi-scale fusion module:

1. Input feature extraction and linear projection: After being processed by the visual encoder tokens, the input features generate a series of feature mappings with dimensions of $T\times D_{0}$, where *T* represents the time steps of the input features and $D_{0}$ is the feature dimension. After the first stage of linear projection, the feature dimension is further increased to T×D.
2. Multi-stage feature extraction: The multi-scale fusion module in the model contains multiple stages of feature extraction. At each stage, the input feature mappings are processed by a state update module, generating new features with dimensions of T×γD, where γ is the amplification factor for feature extraction at that stage. The feature dimensions increase progressively across the stages to extract higher-level features:
  (9) $$X_{i}=f_{{\mathrm{stage}}_{i}}\left(X_{i-1}\right),\;i=1,2,\cdots ,N$$
  where $X_{i}$ denotes the output features at the *i*th stage and $f_{{\mathrm{stage}}_{i}}$ represents the feature extraction function at that stage. By repeatedly extracting and amplifying features across different stages, the model can capture rich feature information.
3. Fusion process and feature concatenation: In the multi-scale fusion module, the features extracted from each stage are fused through a concatenation operation. Assuming that the model has *N* stages, the fused features ($F_{\mathrm{fusion}}$) can be expressed as follows:
  (10) $$F_{\mathrm{fusion}}=\mathrm{Concat}\left(X_{1},X_{2},\cdots ,X_{N}\right)$$
  By concatenating features at different scales, the model obtains a feature map ($F_{\mathrm{fusion}}$) that integrates multi-scale features with dimensions of $T\times D_{v}$, where $D_{v}$ is the dimension of the fused features. The fused features contain rich information from local to global and from low levels to high levels.
4. Feature transformation and linear mapping: The fused feature map ($F_{\mathrm{fusion}}$) undergoes a series of linear mapping operations to further adjust the feature dimensions to meet the requirements of downstream tasks. Initially, instead of directly concatenating the features along the *N* stages as $N\times T\times D_{v}$, the concatenated features are processed to result in effective dimensions of $T\times D_{v}$. During the linear mapping process, the feature dimensions are then increased from $T\times D_{v}$ to $T\times 4D_{v}$. After processing through a nonlinear activation function, the dimensions are reduced back to the original feature dimension:
  (11) $$F_{\mathrm{transformed}}=\mathrm{Linear}\left(F_{\mathrm{fusion}}\right)$$
  Through this linear mapping operation, the feature dimensions are unified, and the features are sufficiently fused both in terms of space and scale.
5. Feature classification and output: After multi-scale feature fusion is completed, the features processed by linear mapping are sent to the classification module (TS mixer classification). The classification module performs the final classification operation on the fused features to obtain the disease detection results.

The design of the multi-scale fusion module is based on the theory of multi-scale feature capture in deep neural networks. It aims to make the model more adaptable and robust when dealing with complex disease scenarios through feature extraction and fusion across different stages. In maize leaf disease detection, the presentation of the disease can vary based on factors such as the leaf’s growth status, lighting conditions, and disease development stages. The multi-scale fusion module ensures the model’s robustness when faced with diverse disease features by extracting and fusing features at different scales. It can accurately detect small lesions at a local scale and also identify extensive diseased areas at a global scale.

#### 3.2.5. State Loss Function

The state loss function proposed in this paper is designed to optimize the performance of the state-space attention mechanism, aiming to more effectively reduce misdiagnoses and missed diagnoses in maize leaf disease detection. Unlike traditional loss functions (such as cross-entropy loss), the state loss function not only focuses on the difference between classification results and true labels but also introduces feature states and spatial relationships, optimizing the spatiotemporal consistency of features. Specifically, the state loss function consists of two parts: classification loss and state loss. The first part of the state loss function is the traditional cross-entropy loss ($L_{CE}$), which ensures the classification accuracy of the model:

(12) $$L_{CE}=-\sum_{i=1}^{N}y_{i}\log\left(p_{i}\right)$$

The second part is the state loss ($L_{state}$), which measures the consistency between state information and spatial features within the model. The state loss calculates the temporal differences between feature states to ensure consistency during the state update process. It is defined as follows:

(13) $$L_{state}=\frac{1}{T}\sum_{t=1}^{T}{∥\tilde{S}\left(t\right)-\tilde{S}\left(t-1\right)∥}^{2}$$

where $\tilde{S}\left(t\right)$ and $\tilde{S}\left(t-1\right)$ represent the state representations at time steps *t* and t−1, respectively, and ${∥\cdot ∥}^{2}$ denotes the Euclidean distance. The state loss ensures smooth state transitions across different time steps, providing better temporal continuity in disease detection. The final state loss function is formed by weighting and summing the classification loss and state loss to create a comprehensive target loss function:

(14) $$L=L_{CE}+\lambda L_{state}$$

where λ > 0 is a balancing parameter used to control the relative importance between classification loss and state loss. We determine the optimal value of the λ parameter through an experimental approach, with specific steps outlined as follows:

First, we use cross-validation to test λ across a series of candidate values, typically starting with smaller values, such as 0.1, 0.5, and 1. We apply each candidate value during the model training process and evaluate the model’s performance on the validation set. By observing how different λ values affect the balance between the classification loss ($L_{CE}$) and the state loss ($L_{\mathrm{state}}$), as well as the overall accuracy and robustness of the model, we can gradually narrow down the range of λ values.

When determining the optimal λ value, we focus on the model’s overall performance on the validation set to ensure that the classification task and state task can work together without conflicting. Through this experimental optimization process, we ultimately select a λ value that achieves a reasonable balance between classification loss and state loss, thereby enhancing the model’s overall performance.

The design of the state loss function is based on the observation that in disease detection tasks, disease features often exhibit spatiotemporal correlations and dynamic changes. A simple classification loss cannot adequately capture the temporal evolution of these features. By introducing the state loss, the state loss function maintains the continuity of state information along the temporal dimension, thereby enhancing the model’s ability to capture the disease progression process.

During optimization, the state loss function ensures effective modeling of spatiotemporal features in the model. The optimization objective is to minimize the loss function (L), which is expressed as follows:

(15) $$\min_{\theta }L=\min_{\theta }\left(L_{CE}+\lambda L_{state}\right)$$

where θ denotes the model parameters. By applying gradient descent to L, the model is able to simultaneously optimize classification accuracy and the spatiotemporal consistency of state information, achieving high accuracy in disease detection across both spatial and temporal dimensions.

### 3.3. Experimental Setup

#### 3.3.1. Hardware and Software Platform

In terms of hardware, we selected a computer equipped with an NVIDIA RTX 3080 GPU. This graphics card features powerful computational capability and efficient parallel processing performance, making it suitable for the training of moderately sized deep learning models. The RTX 3080 not only has up to 12 GB of GDDR6X memory but also supports tensor cores and ray-tracing technology, which makes it excel in image processing and complex computational tasks. This allowed us to perform the large-scale calculations needed to efficiently train the maize leaf disease detection model, thereby improving training speed.

For the software platform, we mainly used Python and combined it with deep learning frameworks such as TensorFlow 2.16 and PyTorch 1.8. TensorFlow, with its powerful distributed computing capabilities, is well-suited to handle large-scale data and complex models. PyTorch, with its dynamic computation graph and flexibility, particularly excels in experimental research and prototype development. In practice, we built the basic model in TensorFlow according to the model’s needs and performance requirements while implementing some complex custom modules and experiments in PyTorch.

#### 3.3.2. Experimental Configuration

Next, we chose the Adam optimizer for parameter updates. The Adam optimizer combines the advantages of Momentum and RMSProp, enabling adaptive learning-rate adjustments and thereby improving convergence speed. We set the learning rate to 0.001 based on previous experimental results, ensuring a balance between convergence speed and avoiding oscillations caused by an excessively high learning rate. During training, we set the batch size to 32, which ensures training efficiency while fully utilizing memory resources. A smaller batch size helps the model generalize better, while a larger batch size can speed up training. Experiments showed that a batch size of 32 effectively balances training speed and model performance for this task.

As for the number of training epochs, we set it to 50. This setting was adjusted based on observations from early experiments to achieve optimal convergence. Too many training epochs may lead to overfitting, so we monitored the validation set’s loss during training to determine the optimal number of epochs. To prevent overfitting, we introduced early stopping. When the loss on the validation set did not significantly decrease over several consecutive epochs, the training was stopped, effectively avoiding overfitting on the training set.

During the experiment, we also used 5-fold cross-validation to evaluate the model’s performance. This method divides the dataset into five parts, with four parts used for training and one part used for validation, rotating five times to ensure that each part has a chance to be the validation set. Cross-validation effectively reduces the randomness caused by data splitting, resulting in more reliable model performance evaluation. This process can be expressed by the following formula:

(16) $$\mathrm{Accuracy}=\frac{1}{K}\sum_{k=1}^{K}{\mathrm{Accuracy}}_{k}$$

In this formula, *K* is the number of folds, and ${\mathrm{Accuracy}}_{k}$ is the accuracy of the *k*-th fold. Through this method, we obtained the model’s performance on different data subsets, allowing for a more comprehensive understanding of the model’s generalization ability and stability.

#### 3.3.3. Baseline

In this study, to comprehensively evaluate the performance of the proposed state-space attention-based maize leaf disease detection model, we selected multiple classical CNN architectures as baseline models for comparison. These baseline models include AlexNet [48], ResNet [49], GoogLeNet [50], EfficientNet [51], and ViT [52].

#### 3.3.4. Evaluation Metrics

During the testing phase, to comprehensively evaluate the performance of the proposed state-space attention-based maize leaf disease detection model, we systematically compared all models. This evaluation process used multiple metrics, such as precision, recall, F1 score, and accuracy. The model was trained and tested on seven distinct categories, including “No Disease”, “Maize Northern Leaf Blight”, “Maize Southern Leaf Blight”, “Maize Smut”, “Maize Head Smut”, “Maize Round Spot”, and “Maize Brown Spot”. These labels correspond to different types of diseases, allowing the model to distinguish between various conditions during training and testing. These metrics not only provide quantitative indicators of model performance but also offer directions for further improvement.

Precision measures the proportion of true positives (i.e., correctly predicted diseases) among all predicted positives. Recall, also known as sensitivity, measures the proportion of true positives among all actual positives. To balance precision and recall, we used the F1 score as a comprehensive metric of model performance. The F1 score is the harmonic mean of precision and recall, striking a balance between the two, especially in cases of class imbalance. The F1 score is calculated as follows:

(17) $$F1=2\cdot \frac{\mathrm{Precision}\cdot \mathrm{Recall}}{\mathrm{Precision}+\mathrm{Recall}}$$

The F1 score directly reflects the model’s overall performance in handling disease detection tasks and effectively evaluates the model’s ability to handle both positive and negative samples. Finally, accuracy is one of the most commonly used performance metrics and represents the proportion of correctly classified samples among all samples. The formula for calculating accuracy is

(18) $$\mathrm{Accuracy}=\frac{TP+TN}{TP+TN+FP+FN}$$

Although accuracy is a useful metric, it can be misleading in cases of class imbalance. Therefore, in disease detection tasks, we usually consider multiple evaluation metrics simultaneously to comprehensively reflect the model’s performance.

## 4. Results and Discussion

### 4.1. Maize Disease Detection Results

The purpose of this experiment is to evaluate the performance of different models in maize disease detection and to verify the effectiveness of the state-space attention mechanism model proposed in this paper. By comparing with common deep learning models (including AlexNet, GoogLeNet, ResNet, EfficientNet, and ViT), the table demonstrates the performance of each model across four evaluation metrics: precision, recall, accuracy, and F1 score.

According to the data in Table 2, the precision, recall, accuracy, and F1 score of AlexNet are around 0.80, showing relatively low performance. This is primarily because AlexNet is a relatively shallow model with fewer parameters and limited feature extraction capabilities, making it challenging to capture complex disease characteristics. GoogLeNet, on the other hand, improves upon AlexNet by increasing the number of parameters and employing the Inception module to perform multi-scale feature extraction, which enhances its precision and recall to 0.85 and 0.83, respectively, with an F1 score of 0.84. ResNet introduces residual connections, addressing the gradient vanishing problem in deep networks and maintaining effective feature transmission across multiple layers. As a result, it achieved better performance in the experiment, with precision, recall, and accuracy reaching 0.88, 0.86, and 0.87, respectively. EfficientNet further optimizes the model architecture by balancing network depth, width, and resolution, allowing for efficient computation while adequately capturing features. This leads to further improvements in all metrics, with precision and F1 score reaching 0.90 and 0.88, respectively. ViT represents the application of transformers in computer vision and leverages self-attention mechanisms to model global features of images effectively, significantly improving the capture of disease characteristics. In the experimental results, its precision, accuracy, and F1 score are all above 0.90, while the recall is slightly below 0.90, at 0.89. The method proposed in this paper, based on the state-space attention mechanism, surpasses all other models in the table across all metrics, with precision, recall, accuracy, and F1 score all reaching 0.94. This superior performance is attributed to the state-space attention mechanism’s ability to model spatiotemporal features effectively. Traditional models such as AlexNet and GoogLeNet mainly rely on convolution operations for local feature extraction, facing limitations in feature fusion. ResNet partially overcomes the issue of gradient vanishing in deep learning through residual connections, enabling effective transmission of features across deep layers. However, convolutional neural networks still face constraints due to their limited local receptive fields, making it difficult to capture global disease characteristics. In contrast, ViT establishes global relationships between image patches through self-attention mechanisms, compensating for the limited local perception capability of convolutional networks. However, ViT’s capacity for modeling feature states along the temporal dimension is limited, which prevents it from fully considering the dynamic changes in disease features across time and space. The method proposed in this paper introduces the state-space attention mechanism, which combines input features with state information, establishing connections across both temporal and spatial dimensions. This enables the model to more accurately identify and differentiate the development dynamics of various diseases. The state-space attention mechanism ensures smooth transitions of state information over time when handling maize leaf disease detection tasks and models the spatiotemporal correlations of disease characteristics. This significantly enhances the model’s ability to capture disease features, ultimately achieving high-precision detection. Multi-level and multi-scale feature modeling and fusion improve the model’s generalization ability and robustness in dealing with complex maize disease scenarios, leading to performance superior to that of other models in the experiment.

### 4.2. Results Analysis

#### 4.2.1. Analysis of Different Diseases

The purpose of this experiment was to analyze the specific detection performance of the state-space attention mechanism-based model proposed in this paper on different types of maize leaf diseases, aiming to verify the model’s generalizability and robustness.

The results in Table 3 indicate that the model achieves a recall of 0.99 for “Maize Northern Leaf Blight”, suggesting a strong detection ability for this disease. Its accuracy and F1 score for this disease reach 0.98 and 0.95, respectively, indicating high recognition precision. This performance can be attributed to the relatively large number of samples for this disease, as well as its distinct characteristics, enabling the model to effectively learn its features. In contrast, the recall and accuracy for “Maize Southern Leaf Blight” are both 0.91, slightly lower than those for other diseases. This suggests that the model may experience some misclassification or omission in detecting this disease, likely due to less distinct features and a smaller sample size, which could limit the model’s ability to learn these characteristics effectively. Additionally, for “Maize Smut” and “Maize Head Smut”, the model achieves high precision of 0.96 and 0.99, respectively, demonstrating its stability and adaptability in recognizing different disease features. The model’s performance on “Maize Round Spot” and “Maize Brown Spot” is also noteworthy. For “Maize Round Spot”, the precision and F1 score are 0.93 and 0.92, respectively, showing strong disease recognition capabilities. For “Maize Brown Spot”, the model achieves a precision of 0.96 and an F1 score of 0.92. Despite the smaller sample size, the model maintains a high detection performance, which suggests that the proposed model demonstrates strong generalization ability, even when handling imbalanced data. Overall, these experimental results show that the state-space attention mechanism-based model performs well across various maize disease types, particularly in cases where sample sizes are large and disease characteristics are distinctive.

#### 4.2.2. Confusion Matrix Analysis

The purpose of this experiment was to evaluate the model’s classification accuracy across different disease categories through confusion matrix analysis, with a particular focus on identifying misclassifications to further assess the model’s performance in maize disease detection.

**Figure 6:**
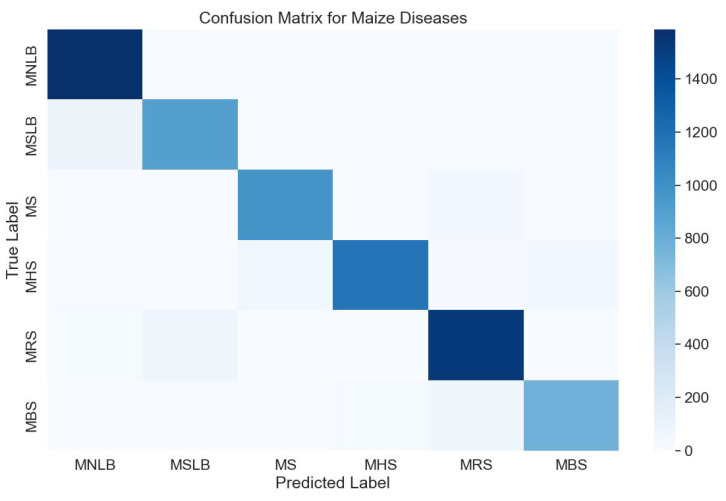
Confusion matrix.

As shown in Figure 6, most disease predictions are concentrated along the diagonal, indicating high accuracy in identifying these diseases. However, the presence of certain off-diagonal elements reveals confusion between specific diseases, especially between “Maize Northern Leaf Blight” (MNLB) and “Maize Round Spot” (MRS). This confusion may stem from the morphological similarities between these two diseases, making it challenging for the model to distinguish between them. Additionally, the confusion matrix highlights the model’s sensitivity to data distribution, as it may exhibit misclassification when sample sizes are small or when disease features are relatively subtle. Through this confusion matrix analysis, we can intuitively identify the model’s shortcomings, providing guidance for further optimization, such as by increasing dataset diversity or enhancing feature extraction methods to improve the model’s discrimination capability.

### 4.3. Ablation Experiments and Discussion

#### 4.3.1. Discussion on Different Attention Mechanisms

The purpose of this experiment was to evaluate the performance of different attention mechanisms in the task of maize leaf disease detection, aiming to verify the effectiveness of the state-space attention mechanism proposed in this paper. Through comparison with standard self-attention and the Convolutional Block Attention Module (CBAM) [53], Table 4 presents the performance of these three attention mechanisms across four evaluation metrics.

According to the data in the table, the standard self-attention mechanism achieves relatively low performance across all metrics, with a precision of 0.74, recall of 0.70, and F1 score of 0.72. This is because standard self-attention, while modeling global features, fails to sufficiently capture the relationships between local features, leading to a limited ability to capture disease details. In contrast, CBAM enhances local feature perception by introducing channel and spatial attention, resulting in improved performance across all metrics, with its precision and F1 score reaching 0.87 and 0.85, respectively. However, while CBAM improves the feature extraction capabilities of convolutional networks through enhanced local modeling, it still lacks the ability to model temporal information effectively. The state-space attention mechanism demonstrated a significant advantage in the experiment, achieving a precision of 0.95 and an F1 score of 0.94. This performance improvement is mainly due to its advancements in spatiotemporal feature modeling. The state-space attention not only performs both local and global spatial feature modeling but also introduces temporal state updates and memory, enabling the model to capture the dynamic process of disease features changing over time. This design mathematically establishes connections along the temporal dimension, allowing the model to accurately model and recognize different developmental stages and state changes of the disease in the maize leaf disease detection task. Consequently, the state-space attention mechanism shows comprehensive performance that is significantly superior to that of both standard self-attention and CBAM.

#### 4.3.2. Discussion on Different Loss Functions

The purpose of this experiment was to evaluate the impact of different loss functions on the performance of the maize leaf disease detection model and to verify the effectiveness of the state-space loss in comparison to traditional cross-entropy loss and focal loss.

As seen in Table 5, different loss functions have a significant impact on the model’s performance in detecting maize leaf diseases. First, when using the cross-entropy loss, the model’s precision, recall, accuracy, and F1 score all range between 0.65 and 0.69, showing relatively low performance. This is because cross-entropy loss, a traditional classification loss function, works by calculating the logarithmic probability difference between predicted values and true labels, maximizing the probability of predicting the correct class. However, when dealing with imbalanced datasets or complex features, cross-entropy loss is susceptible to noisy samples and cannot adequately account for the distribution differences among various classes in the feature space. Therefore, despite performing well in general classification tasks, cross-entropy loss limits the model’s generalization ability in the maize disease detection task, which is characterized by diverse features and imbalanced data, resulting in suboptimal overall performance. In contrast, adopting focal loss significantly improves the model’s performance, with a precision of 0.83 and an F1 score of 0.81. Focal loss builds upon cross-entropy loss by adding an adjustment term that reduces the loss weight of easily classified samples, focusing more on hard-to-classify samples. The mathematical expression for focal loss is

(19) $$L_{\mathrm{focal}}=-{\left(1-p_{t}\right)}^{\gamma }\log\left(p_{t}\right)$$

where $p_{t}$ is the predicted probability and γ is a tunable parameter controlling the focus on difficult samples. This adjustment strategy enables the model to better handle imbalanced data during training, especially by focusing on challenging disease features for detection. Hence, in the maize disease detection task, focal loss effectively improves the model’s ability to recognize various disease types and enhances overall performance. However, since focal loss still relies on independent classification of individual samples to some extent, it fails to fully consider the spatiotemporal correlations between features. State-space loss achieved the best performance in this experiment. By combining state information with feature relationships, state-space loss effectively models the spatiotemporal characteristics of maize diseases. Traditional loss functions usually treat each sample’s classification independently, but state-space loss introduces state information, enabling the model to account for temporal dynamics and spatial associations between samples. This comprehensive modeling strategy allows the model to extract features more effectively and classify diseases more accurately in the maize leaf disease detection task, resulting in higher detection accuracy and stability.

### 4.4. Limits and Future Work

The maize leaf disease detection model proposed in this paper, based on a state-space attention mechanism, performed excellently in experiments but still has some limitations that require further optimization and improvement. First, although the state-space attention mechanism effectively enhances the spatiotemporal modeling capabilities of features, the complexity of the model is relatively high, leading to longer training times and increased demands for computational resources. For practical agricultural applications that need to process large-scale data, further optimization of the model’s efficiency and real-time performance is necessary.

Secondly, although the dataset contains a rich variety of maize disease samples, the variability of certain disease characteristics across different growth stages or lighting conditions may limit the model’s generalization ability in complex scenarios. Future work could consider introducing more diverse data samples or employing transfer learning and data augmentation techniques to enhance the model’s generalization performance. Moreover, the current model primarily relies on the relationship between input features and state information to model the dynamic process of disease development, but it still requires improvement in handling changes in environmental factors and multi-modal information. Future research could explore integrating multi-source heterogeneous data, such as meteorological and soil information, to enhance the model’s capacity for multi-modal information fusion, thereby achieving more precise disease detection and analysis.

Regarding feasibility in practical applications, the current method may require farmers to take multiple photos of the same maize plant at different times to capture the complete progression of the disease. Future research needs to further clarify the number of photos required and whether it is necessary to use a tripod and take photos on sunny days to ensure the model’s stability and accuracy. Additionally, evaluating the feasibility of these requirements in real agricultural environments and optimizing the conditions for photo capture will make the model more suitable for practical use. In conclusion, although the proposed method has made significant progress in maize leaf disease detection, there is still room for improvement in model efficiency, data diversity, and multi-modal information fusion, which will be the focus of future research.

In experiments, the model proposed in this paper showed superior performance in the task of maize leaf disease detection, significantly outperforming other baseline models, such as AlexNet, GoogLeNet, ResNet, EfficientNet, and ViT, in metrics including precision, recall, accuracy, and F1 score, with precision reaching 0.95 and other metrics achieving 0.94. Compared to advanced models such as the baseline models proposed by Zhang et al., MAF-AlexNet, MAF-VGG19, MAF-ResNet50, MAF-DenseNet161, and MAF-GoogLeNet had accuracies of 0.9311, 0.9493, 0.9741, 0.9701, and 0.9509, respectively [3], and the TinySegformer model had a precision of 0.92, recall of 0.90, accuracy of 0.93, mIoU of 0.85, and Dice coefficient of 0.91, with 65 frames per second (FPS) [12]. Compared to these, our method demonstrated superiority in multiple indicators, especially in terms of generalization and efficiency when dealing with different activation functions.

## 5. Conclusions

This paper presents a maize leaf disease detection model based on a state-space attention mechanism, which effectively enhances detection accuracy and efficiency by integrating state information with feature relations for spatiotemporal modeling. Experimental results show that compared to traditional models such as AlexNet, GoogLeNet, ResNet, EfficientNet, and ViT, our model achieved a precision of 0.95, with other key metrics like recall, accuracy, and F1 score at 0.94, surpassing other models. Particularly in comparative experiments of different attention mechanisms and loss functions, the state-space attention mechanism and state-space loss function performed exceptionally well across all evaluation metrics, with precision reaching 0.95 and an F1 score of 0.94. The main contributions of this research include the introduction of a novel state-space attention mechanism, the design of a multi-scale fusion module, and the introduction of a state-space loss function, offering new methods and insights for the development of smart agriculture. Future research will further optimize the model’s efficiency and generalization capability, explore multi-modal information fusion, and provide more comprehensive solutions for disease detection.

## Figures and Tables

**Table 1:** Number of images for different diseases.

Disease	Quantity
Maize Northern Leaf Blight	1596
Maize Southern Leaf Blight	988
Maize Smut	1013
Maize Head Smut	1280
Maize Round Spot	1612
Maize Brown Spot	854

**Table 2:** Maize disease detection results.

Model	Precision	Recall	Accuracy	F1 Score
AlexNet	0.81	0.79	0.80	0.80
GoogLeNet	0.85	0.83	0.84	0.84
ResNet	0.88	0.86	0.87	0.87
EfficientNet	0.90	0.87	0.89	0.88
ViT	0.91	0.89	0.90	0.90
MaizeNet [3]	0.92	0.91	0.91	0.91
Tiny-segformer [12]	0.92	0.88	0.89	0.90
Proposed Method	0.95	0.94	0.94	0.94

**Table 3:** Performance of the model on different maize diseases.

Disease	Precision	Recall	Accuracy	F1 Score
Maize Northern Leaf Blight	0.94	0.99	0.98	0.95
Maize Southern Leaf Blight	0.92	0.91	0.91	0.98
Maize Smut	0.96	0.96	0.91	0.98
Maize Head Smut	0.99	0.91	0.92	0.91
Maize Round Spot	0.93	0.94	0.95	0.92
Maize Brown Spot	0.96	0.90	0.94	0.92

**Table 4:** Ablation experiment with different attention mechanisms.

Model	Precision	Recall	Accuracy	F1 Score
Standard Self-Attention	0.74	0.70	0.72	0.72
Convolutional Block Attention Module	0.87	0.83	0.85	0.85
State-Space Attention	0.95	0.94	0.94	0.94

**Table 5:** Ablation experiment on different loss functions.

Model	Precision	Recall	Accuracy	F1 Score
Cross-Entropy Loss	0.69	0.65	0.67	0.67
Focal Loss	0.83	0.80	0.81	0.81
State-Space Loss	0.95	0.94	0.94	0.94

## Data Availability

The data presented in this study are available upon request from the corresponding author.
